# Analyzing historical and future acute neurosurgical demand using an AI-enabled predictive dashboard

**DOI:** 10.1038/s41598-022-11607-9

**Published:** 2022-05-09

**Authors:** Anand S. Pandit, Arif H. B. Jalal, Ahmed K. Toma, Parashkev Nachev

**Affiliations:** 1 High-Dimensional Neurology, Queen Square Institute of Neurology, University College London, London, UK; 2 Victor Horsley Department of Neurosurgery, National Hospital for Neurology and Neurosurgery, London, UK; 3 UCL Medical School, University College London, London, UK

**Keywords:** Neurological disorders, Computational platforms and environments, Machine learning, Software

## Abstract

Characterizing acute service demand is critical for neurosurgery and other emergency-dominant specialties in order to dynamically distribute resources and ensure timely access to treatment. This is especially important in the post-Covid 19 pandemic period, when healthcare centers are grappling with a record backlog of pending surgical procedures and rising acute referral numbers. Healthcare dashboards are well-placed to analyze this data, making key information about service and clinical outcomes available to staff in an easy-to-understand format. However, they typically provide insights based on inference rather than prediction, limiting their operational utility. We retrospectively analyzed and prospectively forecasted acute neurosurgical referrals, based on 10,033 referrals made to a large volume tertiary neurosciences center in London, U.K., from the start of the Covid-19 pandemic lockdown period until October 2021 through the use of a novel AI-enabled predictive dashboard. As anticipated, weekly referral volumes significantly increased during this period, largely owing to an increase in spinal referrals (p < 0.05). Applying validated time-series forecasting methods, we found that referrals were projected to increase beyond this time-point, with Prophet demonstrating the best test and computational performance. Using a mixed-methods approach, we determined that a dashboard approach was usable, feasible, and acceptable among key stakeholders.

## Introduction

Acute or emergency referrals greatly contribute to the throughput of clinical specialties like neurosurgery, in which a considerable proportion of patients present with life or limb threatening injuries^1,2^, and require prompt transfer and urgent intervention^3^. Now ubiquitous among U.K. neurosurgical centres^4^, electronic referral systems (ERS) can help with on-call triage by transmitting salient patient information between the referring site and the neurosurgical center. Large-scale data can be made available from a hospital’s ERS, offering a reliable indicator of a center’s historical and current service demand. This referral record can be evaluated, providing insights into the volume, type, timing, and geographical distribution of urgent requests and patient transfers. Yet, largely unexplored, is the potential of this repository to make data-driven forecasts regarding future surgical referral volume, enabling departments to more accurately predict and anticipate upcoming service demands.

Of recent and relevant interest to both clinicians and hospital stakeholders is the impact of the Covid-19 pandemic (and associated governmental restrictions) on present and future surgical services^5^. Given the present growing backlog of surgical procedures due to the pandemic^6^, as well as rising acute referral volumes before and after the pandemic^7–9^, the need for tools that can anticipate service demand has become more pressing. They would enable surgical departments to prioritize and allocate resources more dynamically and ensure timely access to treatment^10^. Requisite for this type of work are machine-learning models that can fit and predict time-series data. Time-series models, which are widely used in non-clinical domains^11,12^, have been applied in healthcare to forecast diverse outcomes such as length of hospital stay^13,14^ admissions^15^, bed occupancy^16^ and outbreaks of infection^17^. Several examples also exist within the surgical literature in applying algorithms of this kind in order to predict service demand^18,19^, but few have been trained on acute surgical data^20^. Fewer still justify and compare their choice of forecasting models and parameters, as is recommended for other clinical domains^21^.

Of no less importance in clinical forecasting studies is the demonstration of these results to key stakeholders in order to effect change. Indeed, a lack of data science expertise among clinicians^22^ limits this type of articulation, providing an incentive for the utility of easy-to-use, adaptable visualizations. Healthcare dashboards represent one such method of dynamic visualization, providing an interactive presentation of clinical and service data, gathered in a way that facilitates interpretation and decision-making^23^. They offer staff an efficient means to audit data without requiring much technical ability and can function as a catalyst for quality improvement initiatives. Dashboards have been used in various surgical departments to measure and improve key in-hospital patient outcomes, theater utilization, and intraoperative performance^24–27^. Common to all of these examples is the ability to visually describe data, permitting inference but not prediction. Yet, alongside data curation, dashboards have an opportunity to incorporate more predictive capabilities. Advanced predictive visualizations are currently being employed to aid with the Covid-19 pandemic response effort (https://covid19.healthdata.org^28^) and have the significant advantage of presenting results in ‘real-time’, which means that the models iteratively update as more data is accrued. However, to the best of our knowledge, no such comparable examples exist within surgery or more widely across the sphere of healthcare quality improvement. If such technologies were available, they would be well placed to offer surgical decision-makers not only a snapshot of historical data, but also a contemporaneous, accessible forecast of resource availability and demand. We anticipate that the combination of an interactive dashboard with time-series forecasting capabilities would permit robust, interpretable machine learning predictions about future surgical demand to be made, contextualized with important insights into historical audit data generated by the software’s varied functionality.

To that end, we used a dashboard approach to evaluate acute neurosurgical referrals in a large-volume tertiary neurosciences center in central London, U.K from the start of the Covid-19 pandemic lockdown period until October, 2021. In addition to auditing historical surgical data trends, we compare state-of-the-art time-series forecasting methods against traditional models in order to predict future demand. We hypothesized that neurosurgical referrals would significantly increase during this time period as services returned to pre-Covid levels of capacity. As a secondary objective, and given the novelty of our approach, we employ a mixed-methods analysis to determine whether the dashboard is usable, feasible and acceptable for typical users.

## Methods

### Reporting guidelines

In the absence of a dedicated checklist for time-series forecasting, the study was conducted in accordance with TRIPOD and EPIFORGE guidelines for predictive model development where relevant^21,29^.

### Ethics and regulations

Our retrospective study and use of anonymized referral data was approved by the institutional review board (National Hospital for Neurology and Neurosurgery, London, UK) as a service evaluation (121-202021-CA) with the requirement for informed consent being waived. All methods were conducted in accordance with local and national guidelines and regulations.

### Data collection

**Figure 1:**
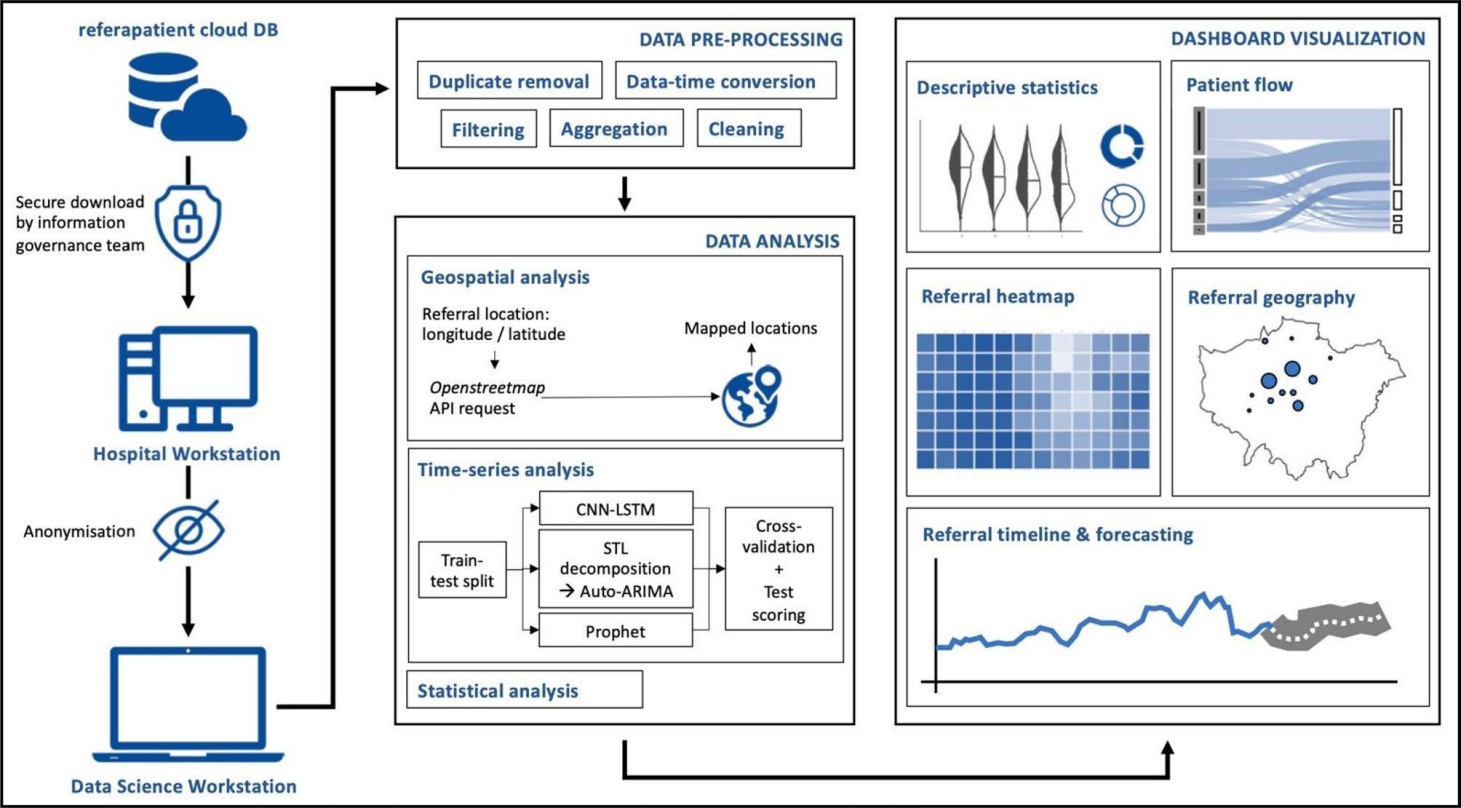
Data acquisition, processing, analysis and visualization pipeline (*CNN-LSTM* convolutional neural network—long short-term memory, *STL* seasonal and trend decomposition using Loess, *ARIMA* automated regression integrated moving average).

A series of data acquisition and processing steps were followed and have been outlined graphically in Fig. 1. Data processing and analysis were performed in Python 3.8.6, using a MacBook Pro (2017, 2.9 GHz, 16 GB RAM). Raw referral data from the center’s cloud-based referral platform (www.referapatient.org) was securely obtained and extracted in *comma separated values* format and downloaded to a hospital workstation before fully de-identifying the data and transferring it to the system aforementioned. *referapatient* is used in the overwhelming majority of neurosurgical centers in the U.K.

### Data analysis and visualization

Data was cleaned and preprocessed to remove erroneous and duplicated entries using *numpy* (v = 1.19.5) and *pandas* (v = 1.2.3) libraries. All Python scripts were run using Visual Studio Code (Microsoft, v = 1.62.0) with results passed into a locally hosted Plotly Dash architecture (v = 1.20.0) built for visualization and data interpretation. Interactive dashboard figures were created using *plotly* (v = 5.3.1). The dashboard allows users to interact with figures through drill-down menus and sliders, which can modify the plots according to categories such as time-interval and diagnosis (Supplementary Video). Dashboard features that would offer high-yield audit and prediction capability were decided in agreement with local faculty, with avenues for further improvement at the feasibility-usability testing stage.

Users could visualize referral data in seven formats: (i) ‘Summary’: a set of figures including violin plots and pie-charts that give a descriptive analysis of the data; (ii) ‘Flow’: a Sankey plot that shows how patients are triaged and on-call decisions are made; (iii) ‘Heatmap’: depicting timing and distribution of referrals in the week; (iv) ‘Geography’: a map of the volume and type of referrals by the referring hospital; (v) ‘Timeline’: a graph of weekly referral volumes; (vi) ‘Time difference’: a statistical comparison of weekly referral volumes between two time periods and (vii) ‘Forecasting’: an artificial-intelligence based time-series analysis to describe future volumes of referral (Fig. 1, Supplementary Video). Whereas the first six formats permit conclusions to be made based on inference, the forecasting section displays predictions of acute surgical demand.

### Time-series analysis

In preparation for time-series analysis, the referral volumes were first sorted into weekly brackets, rather than taking daily volumes, and used as the algorithm variable. This was to compensate for an observed ‘weekend’ effect seen in the daily referral data (Fig. 5A, Supplementary Table 1). Statistical comparisons of weekly referral volumes were implemented through *scipy* (v = 1.6.2). Tests of normality were performed using the Kolmogorov–Smirnov test. If parametric, an independent-samples t-test was applied, otherwise a Mann–Whitney U-test was performed. The choice of forecasting algorithm was limited to options which would: (i) train rapidly while the user is interacting with the dashboard, (ii) automatically parameterize and tune the algorithm without user input, and (iii) handle anticipated seasonality within the data seen after exploratory time-series analysis (Supplementary Results). Three algorithms were felt to meet these criteria: an automated pipeline which combined Seasonal and Trend decomposition using Loess (STL) with an automated regression integrated moving average (Auto-ARIMA) model, a convolutional neural network—long short-term memory (CNN-LSTM) network^30,31^ and Prophet^32^. The implementation of each is now considered in turn (see Supplementary Methods for coding).

ARIMA models are often considered a benchmark model in fields such as econometrics^33^. Here, two adjustments were made to enable automatic hyperparameter tuning and make the model robust to time-series of uncertain length, frame and degree of seasonality. First, a Seasonal and Trend decomposition using Loess (STL) was applied which separated the raw data into seasonal, trend and residual components. Each component was fed into an automated grid search to determine *p, d* and *q* parameters which describe the lag order, degree of differencing and order of moving average respectively. Combinations of parameter values were compared using the Akaike Information Criterion (AIC) in order to determine an optimal set. If the seasonal and trend decomposition failed to enforce stationarity in the trend data (for example, if there were multiple layers of seasonality), the auto-ARIMA step can model the trend, seasonality and residual separately before recomposing the data to forecast.

Deep learning methods can discover and model hidden complexity within data and extract features of interest automatically. CNNs can learn discriminative features by applying a non-linear transformation on time-series data, while LSTM networks mitigate against short-term memory loss via gating methods, to improve learning and information processing within the network. Here, we split the time-series into sub-sequences with 52 “steps” (i.e., 1-year) as the input and one output. This is then split into two sub-samples, each with two targets. This is passed into the convolutional layer, which transforms the subsamples before down-sampling, flattening and passing to a single LSTM layer with 64 neurons. To reduce overfitting, the dropout proportion was set to 30%. The number of filters in the convolutional layer, neurons and dropout proportion were selected following a hyperparameter grid search (~ 30 min). The predicted value was used to iteratively increase the training set for out-of-sample predictions longer than 1 week; thus, the test set is used to progressively fit the model.

Prophet is an open-source library provided by Facebook (https://facebook.github.io/prophet/). Prophet decomposes a time series into four components: growth, yearly and weekly seasonality and holidays, then fits an additive regression model^32^. Growth is modeled as a piecewise linear or logistic growth trend, yearly seasonality is modeled using Fourier series, weekly seasonality is modeled using dummy variables, and holidays are inputted by the user. When modeling, Prophet automatically detects ‘changepoints’ in the trend. In order to identify the changepoint and seasonality prior scale (20 min), we used a grid search hyperparameter tuning (~ 20 min) and specified the lockdown period as a custom “holiday”.

### Evaluation of forecasting algorithm performance

The forecasting model’s performance was evaluated using two methods, with evaluations subdivided into forecast volumes for 1-week, 4-week and 12-week periods. The variety of time frames allows users to assess their ability to predict in the short and long term. First, a ‘blocked’ cross-validation using all available training data (June, 2014 to July, 2021), randomly divided into 5 folds with 15-month time-frames, with a 12-month training window and a 3-month validation window. Second, a train-test approach with a 3-month period (August to October, 2021) withheld from the outset as a testing sample and training data as the year preceding this, i.e., the post-pandemic period. Mean absolute error (MAE), mean absolute percentage error (MPE) and root mean squared error (RMSE) were used as scoring metrics, in addition to the computing time taken to run the algorithm.

### User experience and implementation

In line with other studies in the field of health informatics^34^, we tested user experience using a mixed-methods approach with semi-structured interviews and an electronic questionnaire that incorporated the System Usability Scale (SUS)^35^: a validated tool for usability testing of systems, including healthcare dashboards^36,37^. In addition, we gauged information relating to user implementation through the acceptability of intervention (AIM) and feasibility of intervention measures (FIM)^38^ (Supplementary Methods).

## Results

All figures presented in this section are available as interactive and adjustable graphs within the dashboard platform and can be trialed with the web application which uses synthetic data (Supplementary Material).

### A summary of acute neurosurgical referrals in the post-lockdown period

**Figure 2:**
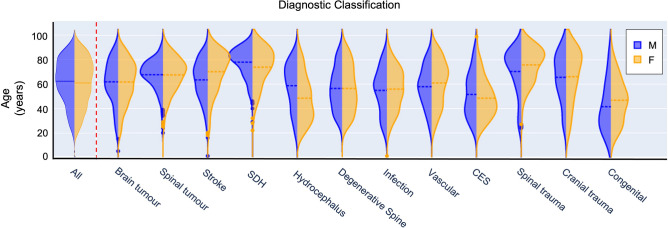
Age and sex distribution of referrals presented by diagnostic classification (*SDH* subdural hemorrhage, *M* male, *F* female).

10,033 acute referrals were made to our neurosurgical center between March, 2020 and October, 2021 (female = 4938, mean age (SD) = 61.1 years (18.8)). As would be expected, the age and gender distribution varied widely according to diagnosis (Fig. 2). For example, patients with a subdural hemorrhage presented with a mean age of 76.8 years (13.6) and male bias (male = 68.3%) as compared to patients classified as being suspected of cauda equina syndrome (mean age = 53.5 years (17.9), male = 43.4%).

**Figure 3:**
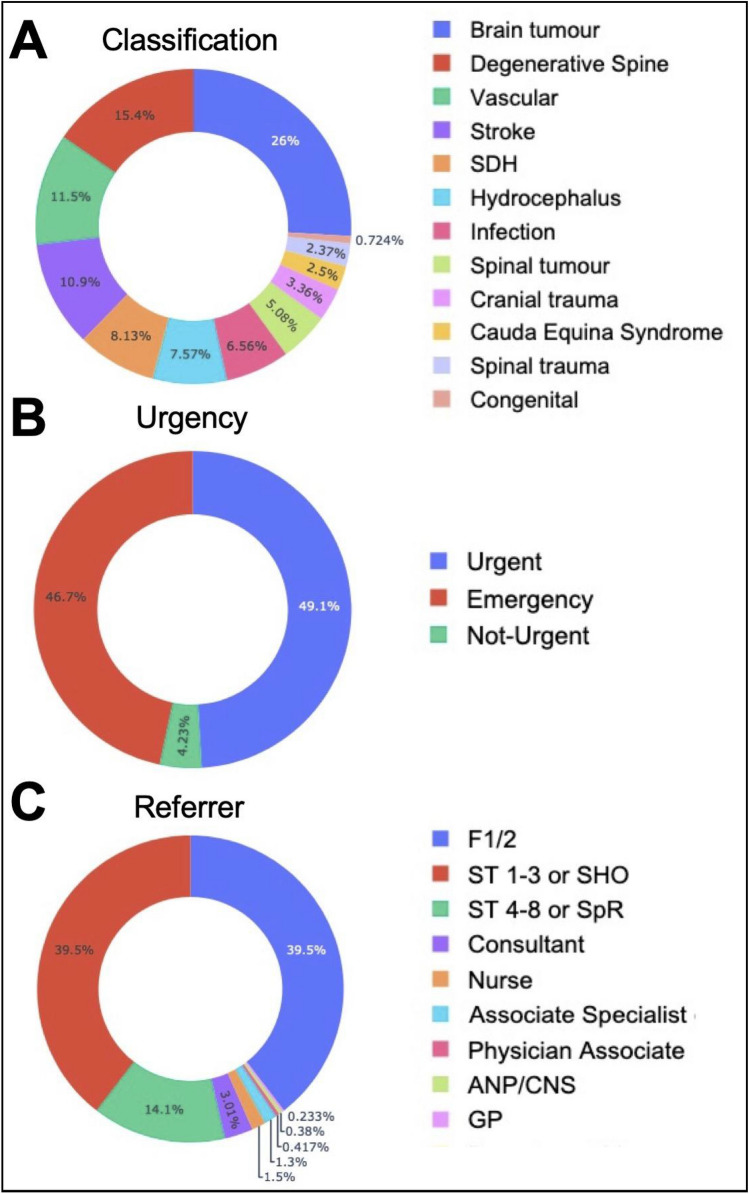
Referral classification. Referral proportion by diagnostic classification (**A**), urgency (**B**), referrer type and seniority (**C**) (*SDH* subdural hemorrhage, *F1/F2* intern/foundation year ½, *SHO* senior house officer, *SpR* specialist registrar/resident, *ANP* advanced nurse practitioner, *CNS* clinical nurse specialist, *GP* general practitioner).

**Figure 4:**
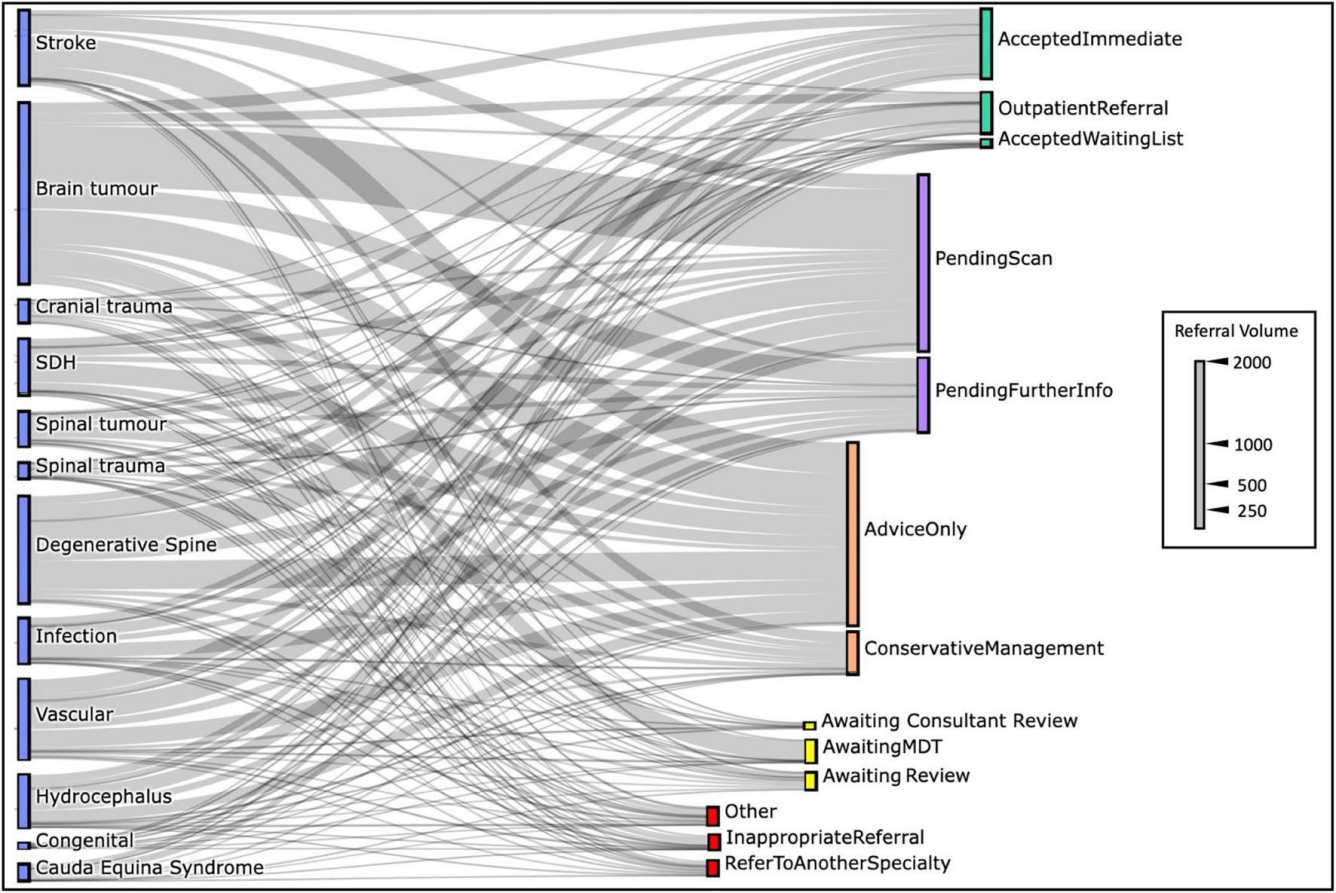
Referral triage decisions. A Sankey plot illustrating decisions made by the on-call registrar/resident sorted by diagnostic classification (blue bars). Triage decisions are grouped according to whether the patient was accepted (green bars), further information was requested (purple bars), advice or conservative management was suggested (orange bars), an additional neurosurgical review was needed to make a decision (yellow bars) or whether the referral was rejected (red bars) (*MDT* multidisciplinary team meeting).

The majority of referrals were classified as brain tumor, degenerative spine or neurovascular diagnoses (Fig. 3A) in line with the center’s main subspecialties. 96% of referrals were stated as ‘emergency’ or ‘urgent’ by the referring team (Fig. 3B) and 79% of referrals were made by a junior registrar or intern (Fig. 3C). In terms of how referrals were triaged, 9.5% of referrals were accepted for immediate hospital transfer, 1% were placed on a transfer waitlist and 6.3% were assigned to outpatient review. 36.4% of referrals required additional clinical or imaging information from the referrer in order to make a triage decision. 32.1% of referrals were completed by triaging to conservative treatment or by offering only advice (Fig. 4).

**Figure 5:**
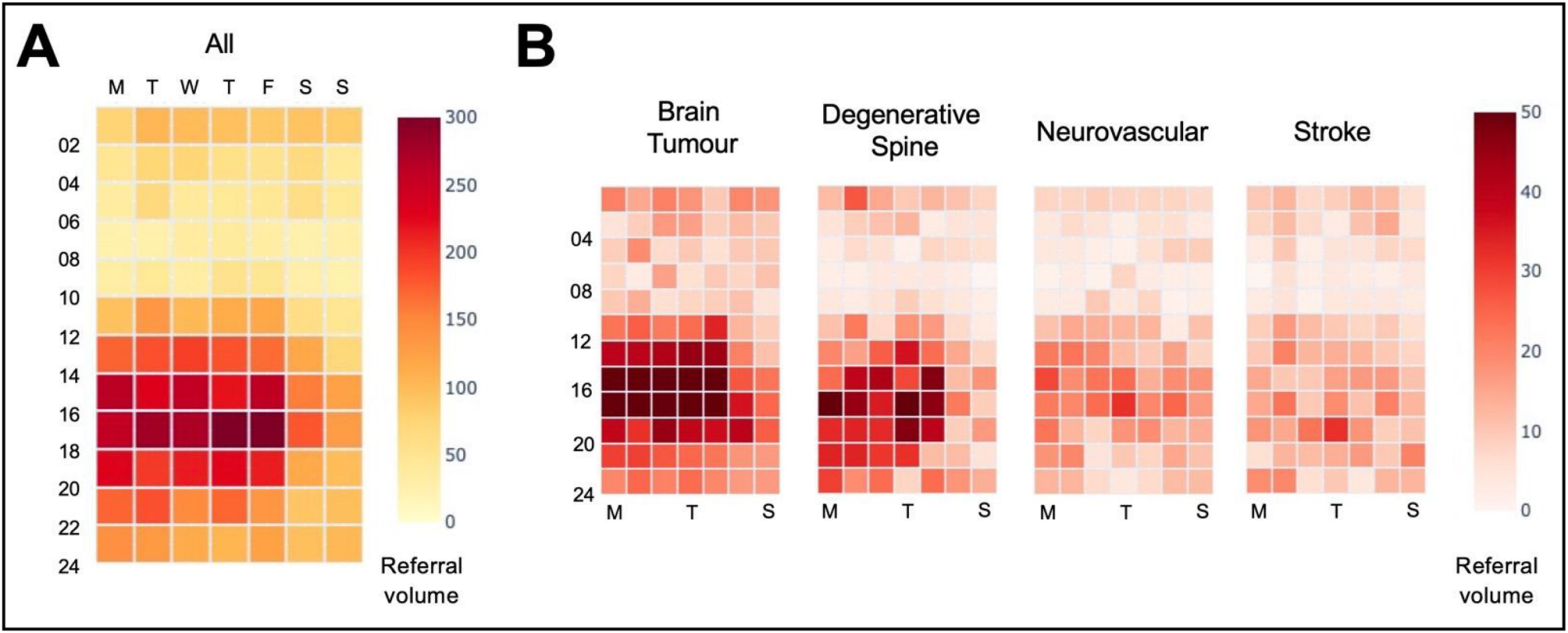
Referral heatmap. Referral volumes sorted by day and time for all (**A**) and for the four highest referring diagnostic categories (**B**).

**Figure 6:**
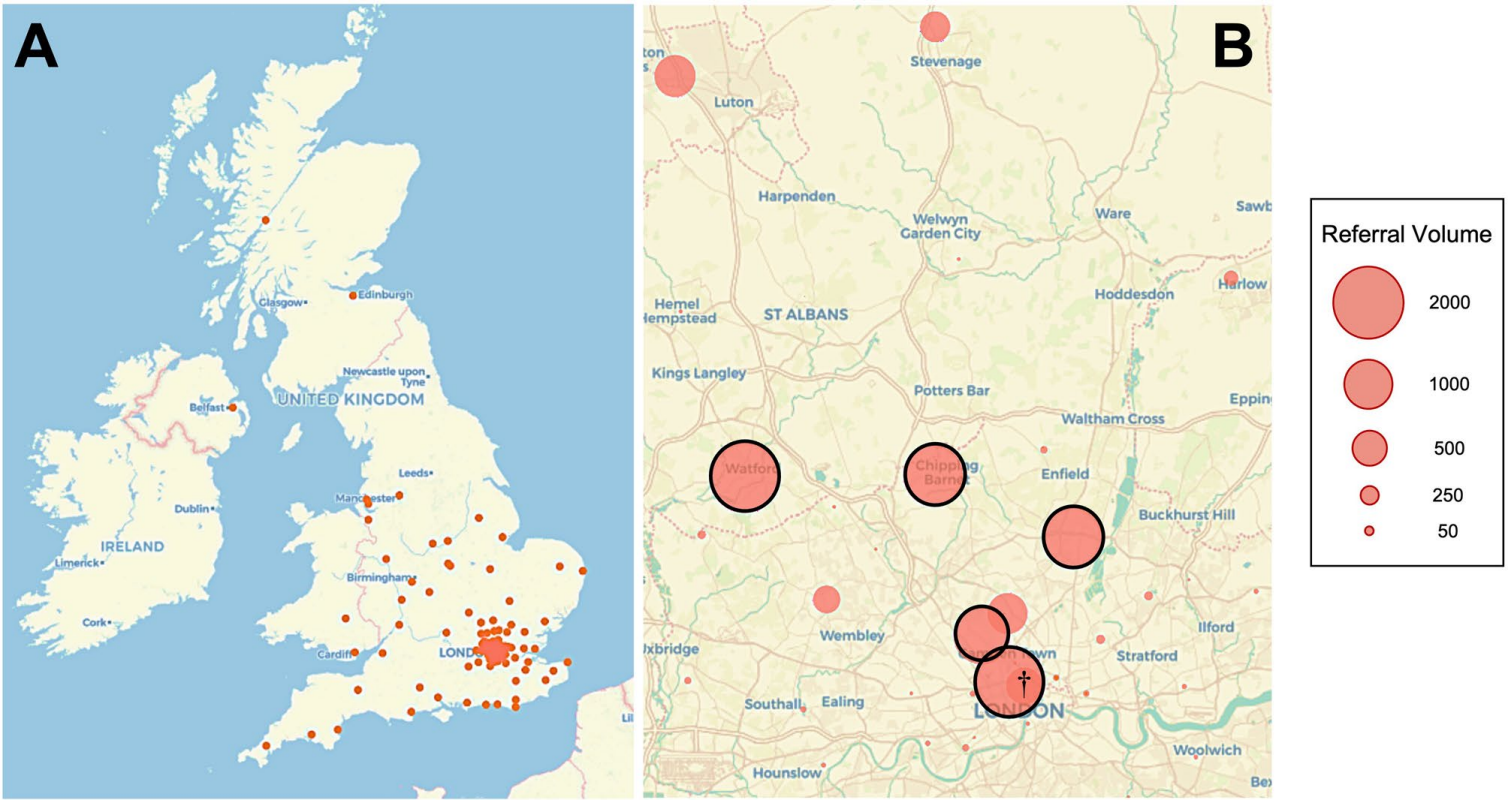
Geographic referral visualization. (**A**) Map of referring sites (red dots) to the neurosurgical center from across the U.K. between March, 2020 and October 2021. (**B**) Northern Greater London referral catchment area with referring sites (red circles) size proportional to referral volume. The five highest volume main referring sites are highlighted with black borders. ^**†**^Denotes the approximate location of the receiving neurosurgical center.

Weekly referral timing was found to be concentrated between 2 and 6 PM on weekdays (Fig. 5A), particularly for brain tumor referrals. Referrals for other high-volume categories such as degenerative spine and neurovascular diagnoses were more evenly distributed but still significantly less over the weekend (Fig. 5B, Supplementary Table 1). During the aforementioned time period, referrals were received from 116 hospital sites and clinical institutions from across the U.K. (Fig. 6A). Five hospitals in the Greater London catchment area accounted for more than 70% of the overall referral volume (Fig. 6B).

### Choice of forecasting algorithm

**Figure 7:**
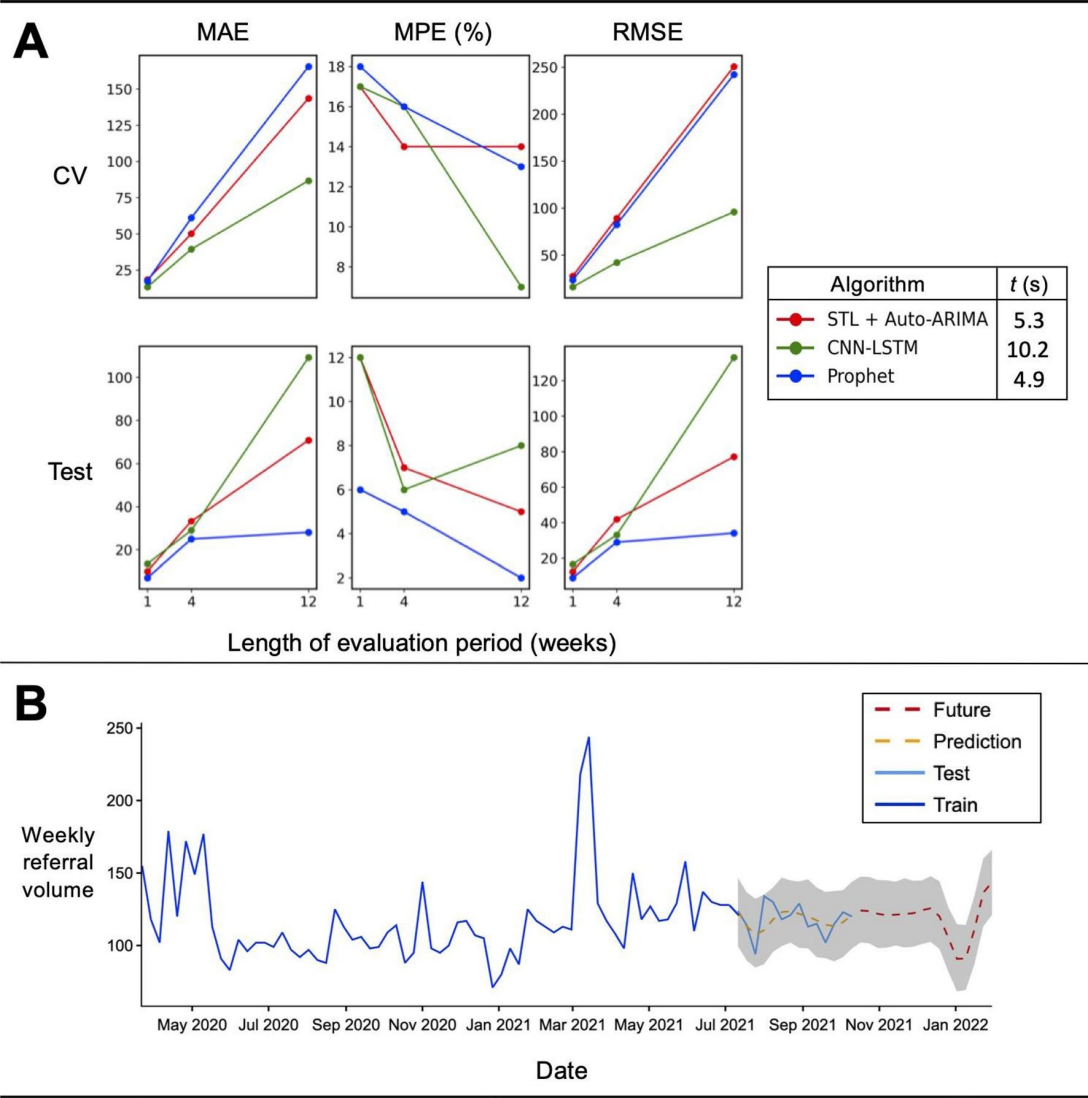
Time-series algorithm performance and prediction of future referral volume. (**A**) Evaluation of time-series forecasting algorithms using fivefold block cross-validation (CV) and train-test split (Test) using mean absolute error (MAE), mean absolute predictive error (MPE) and root mean squared error (RMSE) with 1, 4 and 12 week forecasting periods. Algorithm legend: computational time (*t*) taken to run onefold of each algorithm (*STL* seasonal and trend decomposition using Loess, *CNN-LSTM* convolutional neural network—long short-term memory, *ARIMA* automated regression integrated moving average). (**B**) Timeline of weekly referral volumes plotted since the start of the Covid-19 pandemic with in-sample forecasting (prediction: yellow dashed line) and 12-week out-of-sample forecasting (future: red dashed line) determined by Prophet with 95% confidence intervals shown in gray.

Prioritizing model-fitting time and test performance, Prophet was selected as the dashboard forecasting algorithm of choice (Fig. 7A). Although the CNN-LSTM algorithm demonstrated better performance across cross-validation scoring metrics (mean absolute percentage error (MPE), 1 week = 17%, 4 weeks = 16%, 12 weeks = 7%), it was found to require a longer computational training and fitting time (10.2 s) making it unsuitable under real-world computational constraints. ARIMA models are often considered a traditional benchmark model in forecasting^33^. Here, with the addition of STL and an auto-hyperparameter tuning function, the cross-validation performance was comparable with Prophet, however its test performance was worse (Test-MPE, 1w = 12%, 4w = 7%, 12w = 5%) and was also marginally slower (5.3 s). Prophet had the lowest error rates across all test scoring metrics (Test-MPE, 1w = 6%, 4w = 5%, 12w = 2%), for all time periods and had the fastest computational time (4.9 s).

### Change in referral volumes

Weekly referral volumes were compared between the first 6 months after the announcement of the U.K. Covid-19 pandemic lockdown^39^ and the same time frame after 1 year. There was a significant increase between these periods (Mann–Whitney U (MWU), p < 0.05), mainly driven by an increase in spinal referrals (MWU, p < 0.05) which included spinal trauma, suspected cauda equina syndrome and degenerative diagnoses (Table 1). For this diagnostic group, there were on average 10 additional referrals per week between the early and late Covid-19 periods. Out-of-sample forecasting by all three time-series algorithms using all available training data demonstrated a consistent increasing long-term referral trend (Supplementary Fig. 1, Fig. 7B).

**Table 1:** Median weekly volumes and group-wise differences between the first 6 months of lockdown (March to August, 2020) and the same corresponding months after 1 year.

Diagnosis	Early Covid-19 period median weekly volume	Late Covid-19 period median weekly volume	Difference	p
All	104	121	17	0.02
Cranial*	59	62	3	NS
Spinal**	34	44	10	0.02
Other^†^	8	13	5	< 0.0001

All p-values are Bonferroni multiple comparison corrected following univariate Mann–Whitney U tests.

*NS* not significant.

*Aggregate of cranial trauma, subdural hemorrhage, stroke, brain tumor, neurovascular disease, congenital diseases and hydrocephalus diagnoses.

**Spinal trauma, cauda equina syndrome, degenerative spine and spinal tumor diagnoses.

^†^Central nervous system infection, non-neurosurgical diagnosis).

### Usability, feasibility and acceptability

20 participants were recruited for feasibility testing, including 5 neurosurgical consultants, 12 registrars/residents and 3 members of management or administrative staff. All were blinded to the development of the dashboard. Table 2 lists the average SUS, AIM and FIM scores among participants. An SUS score of 70 or above has previously been defined as a threshold for good usability^34^. In this study, all user groups had mean SUS scores above this benchmark and high mean acceptability (AIM) and feasibility (FIM) scores were also recorded.

**Table 2:** Usability, feasibility and acceptability scores among main user groups.

User group	n	SUS mean (SE)	AIM mean (SE)	FIM mean (SE)
All	20	77.1 (3.0)	4.7 (0.2)	4.6 (0.2)
Registrar/resident	12	78.0 (3.1)	4.8 (0.1)	4.7 (0.1)
Consultant/attending	5	74.2 (6.8)	4.4 (0.4)	4.2 (0.4)
Management and administration	3	78.3 (10.4)	4.9 (0.1)	5.0 (0)

*SUS* system usability scale, scored out of 100, *AIM* acceptability intervention measure, scored out of 5, *FIM* feasibility intervention measure, scored out of 5, *SE* standard error.

Analysis of user feedback explored possible reasons why the dashboard scored well (Supplementary Results, Supplementary Table 2). In brief, users highlighted the figures and interactivity as particularly useful features and felt that the dashboard would be useful to explore referral data, identify current areas for service improvement and suggest future directions for research. The use of time-series forecasting was commented as useful in anticipating service demand. In contrast, users expressed concerns regarding how the dashboard would be hosted and wished for additional functionality to extract and review the data at patient or smaller sub-group levels.

## Discussion

In this study, we found that as anticipated, referral volumes significantly increased between the first 6 months after the Covid-19 pandemic began and a corresponding time period 1 year later. This was consistent with the findings of ElGhamry et al., who discovered increased referral and operative volumes were apparent in the post-wave period as services were restored to pre-pandemic levels^40^. Changes in referral volume at our center were mostly related to an increase in spinal referrals encompassing conditions such as degenerative spine and suspected cauda equina syndrome. We found that spinal activity, in particular, was reduced during the initial pandemic period. This is most likely due to patient avoidance of healthcare services in order to prevent viral transmission, postponing their clinical presentation^41^. Governmental self-isolation advice and lower vehicular traffic (and associated road-traffic injuries) may have also contributed to this reduced case-load^40^. However, outside our own referring catchment, lower spinal referrals did not necessarily translate into fewer spinal procedures^42^. We also observed that time-critical neurovascular referrals such as cerebrovascular stroke were less distributed during the weekend rather than weekdays (Supplementary Table 1), suggesting delays for presentations such as this may be associated with worse outcomes.

Beyond the dates of our testing set (from October 2021), we forecasted that weekly referral volumes would continue to rise over the next year. To evaluate our forecasting functionality, we compared ‘standard’ models of time-series forecasting (STL + ARIMA) against novel methods, including deep learning algorithms, with the stipulation that they could be fitted quickly while the dashboard was in use. Although the combination of CNN and LSTM methods produced the best cross-validation results, it was discovered they were computationally intensive to fit. Similar to previous work^43^, we found that Prophet was the most time-efficient and had comparable cross-validation scores as compared to STL + ARIMA. In the withheld testing sample, Prophet also outperformed both. We acknowledge that other time-series models could have been trialed to achieve better predictive performance. For example, Aravazhi found that hybrid models typically out-performed ‘simple’ models when aiming to forecast elective surgical volumes, although this was not found to be universal for all surgical time-series data. Even so, trials of hybrid or ensemble methods^44^ would be a valuable future direction for our work.

A shortcoming shared by all our forecasting methods was that tuning was performed over a narrow parameter space, implying that there was scope to improve the models with a wider grid search^44^. Nevertheless, our intention was to improve generalizability by reducing hyperparameter optimization time and make the software ‘plug and play’. The Covid-19 period represents a period of uncertainty and volatility, which may not be representative of typical referral patterns. Since model training includes this period, it is possible that the fitted models will be less accurate when referral trends normalize. To compensate, the period of model training is adjustable in the dashboard and both the STL + ARIMA and Prophet pipelines can adjust to time-series trend changes (see “Methods” section). We highlight several other features to reduce overfitting^45^, including the use of dropout layers, dataset splitting into adequate train, validation and test sets, automatic hyperparameter tuning and heterogenous referral data from a wide array of referring centers (Fig. 6A). The use of external datasets would nonetheless be indispensable for confirming model validity and generalizability^45^.

Although there is no shortage of time-series forecasting examples within the recent surgical literature^18,44^, there are few that precisely describe acute referral numbers. Chandrabalan et al. trained a forecasting model using Prophet to estimate the pandemic-related deficit in colorectal cancer referrals and found that their predictions overestimated referral volumes as compared to actual data in the early post-pandemic period^20^. The fact that all algorithms examined in our work had 12-week mean percentage error scores of less than 10 and 15% for testing and cross-validation respectively and that similar trajectories and volumes were forecasted out-of-sample, lends credibility to our predictions (Supplementary Fig. 1). We note these error levels are sufficiently below thresholds described in other literature^46^.

The results in this study were embedded within a dashboard platform to permit accessible visualization and interpretation and enable users with little to no data-science experience to audit referral data using highly interactive features. Referrals could be filtered according to diagnosis, timeframe, demographics, geographical location, decision-making, referrer information and timing. Each of these functions offered important historical insights regarding acute referrals from this period. For example, it was noted that referrals were received at certain hours throughout the week, implying that a re-arrangement of staffing to better match these peak times may improve acute service efficiency. The overwhelming majority of referrals to our center were labeled as ‘urgent’ or ‘emergency’, although few were transferred immediately or placed on a waiting list. The lack of patient information associated with the referral (Fig. 4), could be attributed to staff experience, with junior residents and interns sending most referrals (Fig. 3C). Educational interventions which aim to improve recognition and initial management of neurosurgical presentations would be particularly useful in improving referral quality^47^ and could be directed at high-volume referral centers (Fig. 5B), where a greater impact is likely to be made.

While preliminary, these findings collectively demonstrate our dashboard’s versatility and functionality for drawing critical insights into an acute neurosurgical service as well as suggesting various avenues for future quality improvement and research. We confirmed, using a mixed-methods approach, that key stakeholders who could benefit from this software deemed it as usable, acceptable and feasible. Low-code and no-code development platforms have recently been gaining traction, permitting users to build, test and share applications with minimal expertise^48^. Data dashboards are an example of a low-code platform with modular components that allows people to engage with big-data via an easy-to-use graphical user interface. They facilitate audit and exploratory data analysis without the need for programming or spreadsheets. For developers, each module can be iteratively configured with ease to meet the needs of the user-base, allowing for an almost limitless scope for innovation.

Although dashboards have previously been used to explore healthcare big-data^49,50^, we believe our work is the first to combine surgical large-scale data with time-series machine learning (ML) methods within a dashboard platform. Furthermore, the estimation of future referral volumes could be performed nearly instantly. A key strength of this design is that it considerably reduces the gap in accessibility and technical ability required to understand ML methods and their clinical implementation. Indeed, as two study participants commented: “the new AI tool was really user friendly (R5)” and “it was impressive that AI could be implemented and used (in the dashboard) so easily (R8)”. Within our center, the evidence of greater future service demand has helped instigate processes (outpatient and rota planning) to assist in allocating resources more effectively. While it is not demonstrated within the synthetic platform of this study (Supplementary Results, Video), we highlight the strength in our dashboard being able to update in real-time, at a refresh rate of 1 week, iteratively improving the fitted model and results as more data is collected, although this is not yet fully automated.

We acknowledge other limitations in our study. The accuracy of referral data is highly reliant on the referring doctor and on-call neurosurgical team. Often, there is a change in referral status that is not represented in the record (for example, a pending scan may have been received but the status was not altered) or the specialist working diagnosis was incorrect. Still, some of these inaccuracies are likely to be compensated for once the data has been aggregated. Although several design features were implemented to make the model and software generalizable, further testing on other referral datasets is required to confirm external validity. Furthermore, because our center lacked an existing method for auditing large-volume referral data, there was no control to compare the dashboard's user experience, making it difficult to gauge the relative utility of our software. Separately, our models were designed to predict surgical referrals for a minimum period of 1 week and therefore would not be clinically useful for hyper-acute changes seen on a daily basis.

## Conclusion

Using an AI-enabled predictive dashboard platform, we performed a comprehensive historical and projected analysis of acute neurosurgical referrals made to a large volume tertiary neurosciences center. Using data from during the Covid-19 and post-pandemic period, we highlight important insights gained from these findings and make preliminary suggestions for better allocation of resources and interventions aimed at improving the quality of referrals. In addition to the prospective work aforementioned, there are several opportunities for future development. Setting up an automated pipeline that can accept referral data, fit models and make predictions contemporaneously would contribute toward a widely-held objective of a dynamic, flexible surgical service^51^. Delaying this are several obstacles, including practical concerns regarding streamlining access to multiple information pools and data-regulation issues about how and where this type of clinical dashboard would be hosted. Nevertheless, having a dashboard ‘front-end’ for big-data sets that can both describe and predict, would increase accessibility and stimulate improvements in the quality of patient care.

## Supplementary Information

Supplementary Information.

## Data Availability

The supplementary material contains key portions of the code used. Full code and synthetic data set are available on reasonable request. A version of the dashboard using synthetic data is available on https://referralsdash.herokuapp.com. Clinical data cannot be shared without first obtaining relevant information governance permissions.
