# Supplementary material for: Analyzing historical and future acute neurosurgical demand using an AI-enabled predictive dashboard
Source: Sci Rep. 2022 May 9;12:7603. doi: 10.1038/s41598-022-11607-9 (PMC9084272; doi:10.1038/s41598-022-11607-9)
Supplement: Supplementary file 1 — Supplementary Information. [file 41598_2022_11607_MOESM1_ESM.pdf]

An AI-enabled predictive analytics dashboard for acute neurosurgical referrals

Supplementary Document

Software Demonstration

A trial dashboard using synthetic data can be accessed on: https://referralsdash.herokuapp.com/ via a desktop web browser. Please note it can take up to a minute for the dashboard to load on some internet browsers.

A video demonstrating the functionality of the dashboard presenting the data outlined in this manuscript is available on https://youtu.be/Th2vsCpLHbI

Supplementary Methods

Python libraries and dependencies

The following dependencies were used in the creation of this dashboard (see code snippet below)

<CODE>

```
'pandas', 1.2.3
'numpy', 1.19.5
'matplotlib.pyplot', 3.4.1
'scipy', 1.6.2
'plotly', 5.3.1
'dash', 1.20.0
'dash_core_components', 1.16.0
'dash_html_components', 1.1.3
'requests', 2.25.1
'statsmodels', 0.11.0
'prophet', 1.0.1
'pmdarima' 1.81
'tensorflow' 2.4.1
```

Data pre-processing

Following anonymisation, referral data was uploaded as a *pandas* data-frame. Redundant columns, duplicates and erroneous entries were removed, and all dates and times were transformed to python date-time data-types for further manipulation. Specialist working diagnoses are designated by the on-call neurosurgical registrar when receiving the referral and include a total of 138 different options. The diagnosis is based on the information received at the point of the referral and may be modified as further information is shared or after senior review. Specialist diagnoses were aggregated into 13 primary diagnostic categories: brain tumour, cauda equina syndrome, congenital, subdural haematoma, cranial trauma, degenerative spine, hydrocephalus, infection, spinal trauma, stroke, neurovascular and 'not neurosurgical' (Supplementary Appendix).

<CODE>

```
#Upload anonymised file - either saved as .csv or .pkl

df_all = pd.read_pickle(filename)

#Drop duplicates
```

```
df_all.drop_duplicates(inplace=True)

#Drop redundant columns
df_all.drop(columns = ['Referring Doctor Name','Bleep or Telephone No','MobileNo','Subsequent Doctor Grade Name','Subsequent Bleep Number','Subsequent Mobile No','Subsequent Dr Email Address','Subsequent Consultant Email Address'], inplace = True)

#Transform date-time entries to datetime datatype
df_all = transform_to_datetime(df_all, 'Referral Time')

#Convert specialist working diagnosis into primary diagnostic classification based on diagnosis table - see Appendix table
diagnosis_table = pd.read_csv('diagnoses_table.csv', low_memory=False)
df_all = add_classification_level(df_all, diagnosis_table,
                                  'Primary Classification')

## RELEVANT PROCESSING FUNCTIONS

def match_classification(diagnosis_table, classification_level,
                         diagnosis):
       diagnosis_level = diagnosis_table[
       diagnosis_table['Specialist working diagnosis'] ==
       diagnosis][classification_level]
       if (len(diagnosis_level.values) > 0):
       return diagnosis_level.values[0]
       return 'no_match'

def add_classification(input_df, diagnosis_table, classification_level):
       df_copy = copy.deepcopy(input_df)
       partial_func = partial(match_classification, diagnosis_table,
                         classification_level)
       df_copy[classification_level] = df_copy[
       'Specialist Working Diagnosis'].apply(partial_func)
       return df_copy

def transform_to_datetime(df, time_col):
       copy = df.copy()
       copy[time_col] = pd.to_datetime(copy[time_col], dayfirst=True)
       return copy
```

Geographical information

Using the name of the referring site, an application programming interface (API) request is made to *openstreetmap.org* to derive the latitude and longitude of referral site locations. This location data is then cached and parsed to a geographical plotting function.

<CODE>

```
##API REQUEST TO GENERATE LATITUDE AND LONGITUDE CO-ORDINATES

def placemaker(df_all):

       #Parse and sort dataframe
       geocount = df_all
       geocount = geocount.groupby(by=['Primary Classification','Referring
Hospital'])[['Age']].count().unstack(level=0)
       geocount.columns = geocount.columns.droplevel()
       geocount.fillna(value=0,inplace=True)
       geocount['total'] = geocount.sum(axis=1)
```

```
        geocount.reset_index(inplace = True)

        #Generate empty columns to fill location data in
        geocount['add'] = 0
        geocount['lon'] = 0
        geocount['lat'] = 0
        geocount = geocount.sort_values(by = 'total', ascending = False)
        geocount.reset_index(drop=True, inplace=True)

        #Generate list of unique hospitals from dataframe
        hosplist = geocount['Referring Hospital'].unique()
        hosplist = hosplist.tolist()

        #For each unique hospital, perform an API request
        for i,v in enumerate(hosplist):

        address = v
        url = 'https://nominatim.openstreetmap.org/search/' + urllib.parse.quote(address)+'?format=json'
        response = requests.get(url).json()
        geocount.loc[i,['add', 'lon', 'lat']] = [address,response[0]["lon"],response[0]["lat"]]

        #Create seperate dataframe to save location data to cache
        locmatch = pd.DataFrame()
        locmatch['Referring Hospital'] = geocount['add']
        locmatch['lon'] = geocount.lon
        locmatch['lat'] = geocount.lat
        locmatch.to_csv('locmatch2.csv')

        return geocount, hosplist, locmatch

##GENERATE GEOGRAPHICAL FIGURE

def geospatial(df, date1, date2,classification):

        #select data by time
        geocount = single_period(df, date1, date2)

        #filter df by primary classification and sort
        if classification != "all":
           geocount = geocount[geocount['Primary Classification'] == classification]

        geocount = geocount.groupby(by=['Primary Classification','Referring 
Hospital'])[['Age']].count().unstack(level=0)
        geocount.columns = geocount.columns.droplevel()
        geocount.fillna(value=0,inplace=True)
        geocount['total'] = geocount.sum(axis=1)
        geocount.reset_index(inplace = True)
        geocount = geocount.sort_values(by='total', ascending=False)
        geocount.reset_index(drop=True, inplace=True)
        geocount = geocount.merge(locmatch, on='Referring Hospital')

        #create figure, can be scaled by color or size. Center is the receiving hospital
        fig5 = px.scatter_mapbox(geocount,
                             lat="lat",
                             lon="lon",
                             hover_name="Referring Hospital",
                             hover_data=["total"],
                             zoom=9,
                             height=300,
                             size=geocount.total,
```

```
                                  size_max=40,
                                  color="total",
                                  center={
                                  'lat': 51.6,
                                  'lon': -0.26
                                  },
                                  opacity=0.7)

        #update layouts
        fig5.update_layout(mapbox_style='carto-positron')
        fig5['data'][0]['showlegend'] = False
        fig5['data'][0]['name'] = 'Referring Site'
        fig5.update_layout(margin={"r": 0, "t": 0, "l": 0, "b": 0})
        fig5.update_layout(autosize=True, width=800, height=800)

        return fig5


## RELEVANT PROCESSING FUNCTIONS

def single_period(df, date1, date2):
        return df[(df['Referral Time'] >= date1) & (df['Referral Time'] < date2)]
```

Implementation of time-series forecasting models

Three forecasting algorithms were trialled in this work: an automated pipeline which combined Seasonal and Trend decomposition using Loess (STL) with an automatic Autoregressive Integrated Moving Average (Auto-ARIMA) model, a Convolutional Neural Network - Long Short-Term Memory (CNN-LSTM) network and Prophet. In this section we describe how each model was implemented.

Supplementary Table 1. Median weekday and weekend volumes.
All referrals and the four highest referring categories are shown. p values shown are Bonferroni multiple comparison corrected following univariate Mann-Whitney U tests. (NS = not significant)

Diagnostic Classification	Median weekday volume	Median weekend volume	p
All	34.0	17.5	<0.0001
Brain tumour	6.8	3.5	<0.0001
Degenerative spine	4.6	2.0	<0.0001
Neurovascular	2.4	2.0	0.06
Stroke	2.2	2.0	NS

STL + ARIMA

We performed an exploratory analysis of the time-series using auto-correlation and partial auto-correlation plots in combination with augmented Dickey-Fuller testing to determine the degree of stationarity in the data and assist in defining initial parameters for seasonal decomposition and upper and lower parameter limits for the auto-ARIMA grid search.

<CODE>

```python
### STL/Auto-ARIMA model
#Run EDA on weekly time-series first to manually check seasonality

#Set variables
res = []

#STL period corresponds to expected seasonality. 4 chosen to reflect monthly seasonal changes.
##Also can use 52 for yearly or 26 for 6-monthly seasonality
period = 4

#How long into future/out-of-sample to make forecast
future = 0
#95% Confidence interval
confidence = 0.05

#STL decomposition with default parameters and period - can be further tuned using grid search
res = STL(df, period = period, robust = False).fit()

#Seasonal auto-ARIMA, stepwise can be changed to True for more thorough grid search. Upper and lower
limits regarding p, q, d determined by initial exploratory analysis of data set
smodel = pm.auto_arima(res.seasonal,
                      start_p=0, max_p=5,
                      start_q=0, max_q=5,
                      seasonal=False,
                      stepwise = False,
                      start_d=0, max_d=5,
                      trace=False, error_action='ignore');

#Trend auto-ARIMA
tmodel = pm.auto_arima(res.trend,
                      start_p=0, max_p=5,
                      start_q=0, max_q=5,
                      seasonal=False,
                      stepwise = False,
                      start_d=0, max_d=5,
                      trace=False, error_action='ignore');

#Residual auto-ARIMA
rmodel = pm.auto_arima(res.resid,
                      start_p=0, max_p=5,
                      start_q=0, max_q=5,
                      seasonal=False,
                      stepwise = False,
                      start_d=0, max_d=5,
                      trace=False, error_action='ignore');

#Modelling seasonality
modelsea = SARIMAX(res.seasonal, order = smodel.order, seasonal_order= smodel.seasonal_order).fit()

#If Auto-ARIMA fails then use simple differenced d=1 model for trend
try:
       modeltrend = ARIMA(res.trend, order = tmodel.order, freq=interval).fit()
except:
       modeltrend = ARIMA(res.trend, order = (0,1,0), freq=interval).fit()

#Modelling residual
modelres = ARIMA(res.resid, order = rmodel.order, freq=interval).fit()

#Forecasting and recomposition
forecast_season  = modelsea.forecast(future, alpha=confidence)
```

```
forecast_trend, std_err_trend, confidence_int_trend = modeltrend.forecast(future, alpha=confidence)
forecast_resid, std_err_resid, confidence_int_resid = modelres.forecast(future, alpha=confidence)
forecast_final = forecast_season + forecast_trend + forecast_resid
conf = confidence_int_trend + confidence_int_resid
```

CNN - LSTM

<CODE>

```
###CNN-LSTM implementation

#Relevant imports
from tensorflow.keras.models import Sequential
from tensorflow.keras.layers import LSTM, Dense, Flatten, TimeDistributed, Conv1D, MaxPooling1D

# define input sequence from dataframe
sequence = df['all'].to_list()

# Set number of steps, keep even
n_steps = 52

# split into an array of subsequences, X = input
X, y = sequence_split(sequence, n_steps)

features = 1
n_seq = 2

# divided subsequence into 2 subsamples
n_steps2 = n_steps/2

# reshape input data for CNN layer
X = X.reshape((X.shape[0], n_seq, n_steps2, features))

# set up sequential stack model
model = Sequential()

#CNN layer with 64 output filters, kernel size corresponds to length of convolutional window. Input
shape must match shape from reshape step
model.add(TimeDistributed(Conv1D(filters=64, kernel_size=1, activation='relu'), input_shape=(None,
n_steps2, n_features)))

# Down samples by pool size
model.add(TimeDistributed(MaxPooling1D(pool_size=2)))

#Flatten to single 1D vector
model.add(TimeDistributed(Flatten()))

#Single LSTM layer with 64 neurons
model.add(LSTM(64, activation='relu'))

#NN dense layer
model.add(Dense(1))

#ADAM optimisation using mse as a cost function
model.compile(optimizer='adam', loss='mse')
model.fit(X, y, epochs=500, verbose=0)

## RELEVANT PROCESSING FUNCTIONS

def sequence_split(timeseries, n_steps):
```

```
        #Prepare list variables
        X, y = list(), list()

        for i in range(len(timeseries)):

        # find index at sequence end
        end_index = i + n_steps

        # stop code if has gone past total length of sequence
        if end_index > len(timeseries)-1:
            break

        # divide sequence into subsamples
        sub_x, sub_y = timeseries[i:end_index], timeseries[end_index]
        X.append(sub_x)
        y.append(sub_y)

        return np.array(X), np.array(y)
```

Prophet

<CODE>

```
### Prophet implementation

#Specify dataframe and convert to prophet input

prophetdf = df.reset_index()
prophetdf.columns = ['ds', 'y']

#Specify weeks to predict
prediction = 1

#Specify lockdown period
lockdown = pd.DataFrame({
        'holiday': 'lockdown',
        'ds': pd.to_datetime(['2020-03-23']),
        'lower_window': 0,
        'upper_window': 84,
        })

#Set model parameters. Note data is already in weekly format.
model = Prophet(yearly_seasonality=True,
              weekly_seasonality=False,
               daily_seasonality = True,
              seasonality_mode='additive',
              interval_width=0.95,
              changepoint_prior_scale= 0.05,
              seasonality_prior_scale= 0.1,
              holidays = lockdown)

#Fit model
model.fit(prophetdf)
future = model.make_future_dataframe(periods=prediction,freq='W')

#Make predictions
forecast = model.predict(future)
```

[redacted]

Usability, acceptability and feasibility

This study employed a mixed-method design to assess dashboard usability, acceptability and feasibility. Participants were recruited from the local neurosurgical centre through mailing lists and were included if they had an adequate experience of using the electronic referral system (> 6 months). Participants were excluded if they were aware of the development of the dashboard.

In each testing session, a demonstration of the dashboard's capabilities were shown (~ 10-minutes). As an example which would simulate a typical service evaluation, participants were shown how to use features to audit a particular diagnostic category or time-period. Using a think-aloud protocol, participants were invited to explore the functions of the dashboard independently, after which they completed an electronic questionnaire that incorporated three validated instruments: the System Usability Scale (SUS), Acceptability of Intervention Measure (AIM) and Feasibility of Intervention Measure (FIM) adapted for use. The SUS asks participants to respond to a set of 10 statements using a 5 point Likert scale, with a composite score above 70 defined as "good" usability.

In each of the AIM and FIM scales, participants were presented with 4 statements in reference to the 'intervention' (dashboard) and asked to rate these according to a 5-point Likert Scale. These statements have been previously assessed for substantive and discriminant content validity [3]. Two white-box questions were also incorporated into the questionnaire: "Which aspects or features of the dashboard did you find useful?" and "Do you have any suggestions for improving the dashboard?". The questionnaire has been outlined in full in the Supplementary Appendix.

Web application and synthetic data set

A trial version of the dashboard was hosted using Heroku (www.heroku.com), an online service allowing developers to deploy, manage and scale applications. A synthetic data set was created by taking the original anonymised data set and scrambling demographic and clinical variables, while keeping frequency of aggregate diagnostic classes and outcomes the same. Referral locations were shuffled and replaced with names and locations of English Premier League football stadiums to preserve referral site anonymity.

Supplementary Results

Supplementary Figure 1.
Out-of-sample one-year referral projections using all three forecasting algorithms trained on all available data.

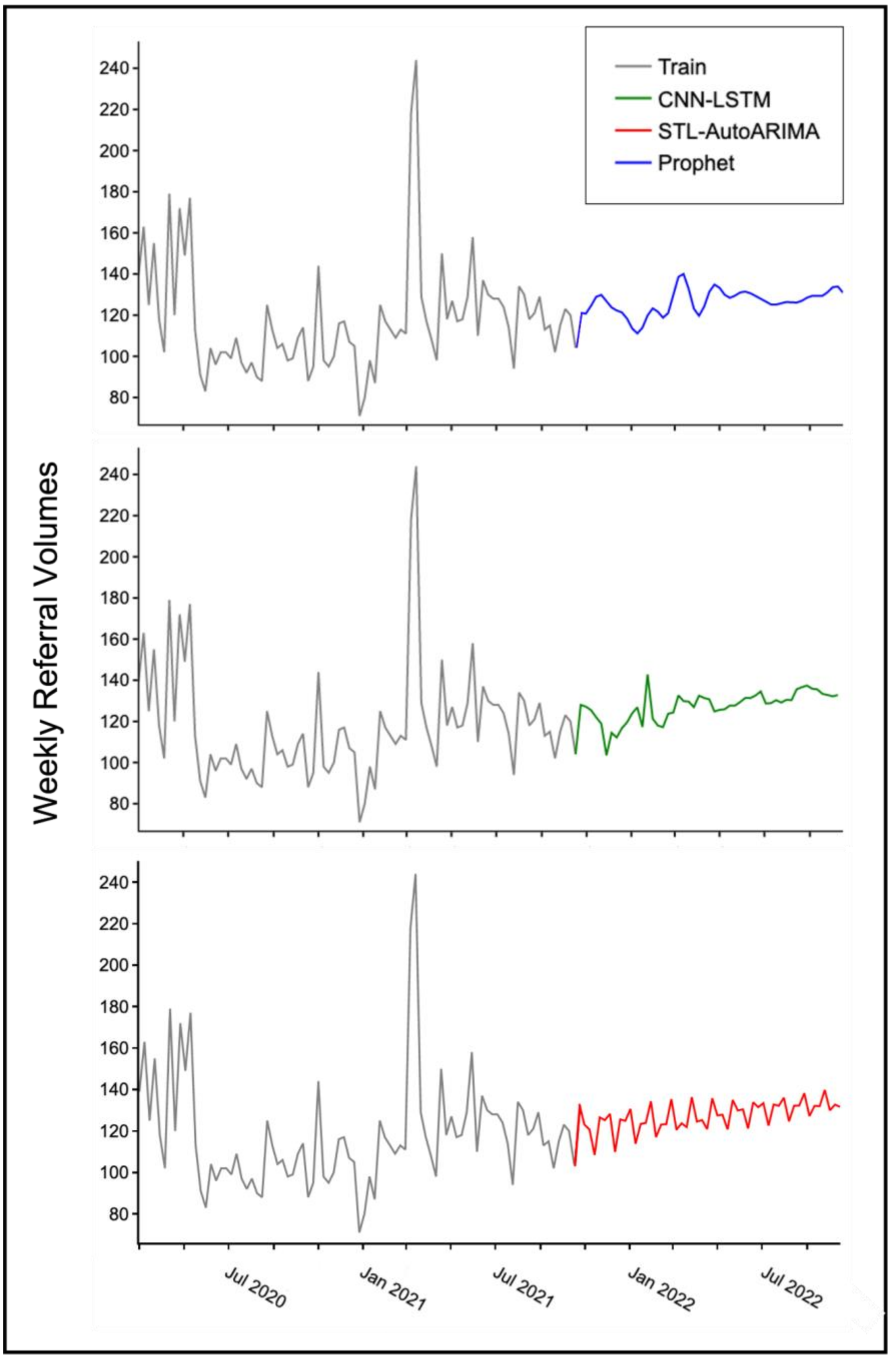

User experience and implementation

Analysis of coded user feedback explores possible reasons why the dashboard scored well (Supplementary Table 2). Many users highlighted the 'clarity', 'usefulness' and 'variety' of graphs and figures [M1, C3, C4, R1, R6, R7, R10, R12]. Others commented on dashboard interactivity [R6, R8], in particular the use of drill-down features as being particularly positive. Some users found that the dashboard would help with auditing and research. In particular they found that it gave important insights 'into previously inaccessible big-data' [R1], that it highlighted 'areas of improvement for staff allocation' [C3], suggested 'directions for more focused audit and research' [C5] and that it demonstrated 'why we need to liaise with local referring sites' [R3]. A few users commented on the AI implementation and time-series forecasting functions stating that it would be 'useful in anticipating demand' [R1], and that it 'could be implemented easily' [R8] but 'unsure how it would be applied day-to-day' [R12]. Some users did express concerns about dashboard access in the department [C1, C2], whereas others thought there should be additional functionality to 'export data' or review it in more detail [R2, R11].

Supplementary Table 2. User feedback and interview responses

**Role**	**Code**	**Which aspects or features of the dashboard did you find most useful?** (Italics = verbal feedback during think-aloud protocol)	**Do you have any suggestions for improving the dashboard?** (Italics = verbal feedback during think-aloud protocol)
Management and Administration	M1	Useful graphs, gives important insights into acute neurosurgical data	/
Management and Administration	M2	*Will be useful in helping understand acute patient flow such as for MSCC (Metastatic spinal cord compression)*	/
Management and Administration	M3	*More audit work should be done like this. Easy to use*	/
Neurosurgery Consultant	C1	*Will be very useful to understand the [acute neurosurgical] service*	*Need to know when it can be accessed by whole department*
Neurosurgery Consultant	C2	Comprehensive. *[Time-series] is interesting. Very useful*	Needs better access
Neurosurgery Consultant	C3	Heatmaps highlight areas of improvement for staff allocation/resources. *Will help make on-call burden easier*	
Neurosurgery Consultant	C4	Beautiful figures	No
Neurosurgery Consultant	C5	Useful at suggesting directions for more focused audit and research	/
Neurosurgery Registrar	R1	Useful figures, can give insight into previously inaccessible big data. Forecasting will be useful at anticipating demand	/
Neurosurgery Registrar	R2	Really useful	More granular outcome data needs to be available
Neurosurgery Registrar	R3	*Shows why we need to liaise with local referring sites [in reference to geospatial figure]*	/
Neurosurgery	R4		/

Registrar			
Neurosurgery Registrar	R5	The new AI tool was really user friendly and I'm excited to see its practical use in research.	/
Neurosurgery Registrar	R6	Nice varied visuals and interaction	/
Neurosurgery Registrar	R7	Very clear figures	/
Neurosurgery Registrar	R8	Instant drill-down and interactivity. *Impressive that AI could be implemented and used so easily [in reference to forecasting]*	*Some functionality (date/time changers) was a bit slow*
Neurosurgery Registrar	R9	*[in reference to geospatial figure] could help improve in determining which sites send poor referrals and how patient transfers could be improved*	/
Neurosurgery Registrar	R10	Nice graphs	Pending information is ambiguous
Neurosurgery Registrar	R11	Excellent dashboard!	Needs ability to download or export data
Neurosurgery Registrar	R12	Highly visual. *You get a good idea of where referrals are coming from. Saves time in looking at spreadsheets*	*Unsure where the AI will be used on a day to day level*

Supplementary References

1. Box, G. E. P., Jenkins, G. M., Reinsel, G. C. & Ljung, G. M. *Time Series Analysis Forecasting and Control*. (John Wiley & Sons, Inc., Hoboken, New Jersey, 2016).
2. Taylor, S. J. & Letham, B. Forecasting at Scale. *Am Statistician* **72**, 37–45 (2018).
3. Weiner, B. J. *et al.* Psychometric assessment of three newly developed implementation outcome measures. *Implement Sci* **12**, 108 (2017).

Supplementary Appendix

Appendix 1. Diagnostic classification.

Specialist working diagnoses typically made by on-call neurosurgical registrar, aggregated into diagnostic classes for further analysis. Where a working diagnosis was fitting more than one class, the most likely class was used.

Specialist working diagnosis	Primary Classification
Brain stem tumour	Brain tumour
Intrinsic cerebellar tumour	
Intrinsic supratentorial tumour	
Brain metastases	
Brain tumour	
Brain tumour: other	
Brain tumour: recurrent	
Extra-axial posterior fossa tumour	
Extra-axial supratentorial tumour	
Intraventricular tumour	
Acute hydrocephalus: tumour	
Pituitary fossa tumour	
Pituitary lesions	
Cauda Equina Syndrome	Cauda Equina Syndrome
Cauda equina syndrome: MR confirmed	
Cauda equina syndrome: suspected	
Cauda equina syndrome/ Acute spinal cord compression	
Congenital abnormality	Congenital
Congenital problem	
Chiari or other hindbrain anomalies	
Congenital spinal lesions of spinal dysraphism	
Congenital cranial lesions	
traumatic brain injury	Cranial trauma
Extradural haematoma	
Extradural Haematoma	
Head Injury	
Head injury: other	
Penetrating Head Injury	
Brainstem injury	
Diffuse axonal injury only	
Posterior fossa injury	
Traumatic intracerebral haemorrhage/contusion	
Traumatic intraventricular haemorrhage	
Traumatic subarachnoid haemorrhage	
Skull fracture only	
Acute Subdural Haematoma	SDH
Acute subdural haematoma	
Chronic subdural haematoma: bilateral	
Recurrent chronic subdural haematoma: bilateral	
Chronic subdural haematoma: unilateral	
Recurrent chronic subdural haematoma: unilateral	
Chronic Subdural Haematoma	
Chronic subdural haematoma	
Subdural hygroma(s)	
Atlanto-axial (C1/C2) degeneration	Degenerative Spine
Cervical degeneration: disc herniation	
Cervical degeneration: neck pain	
Cervical degeneration: stenosis	
Lumbar degeneration: back pain	
Lumbar degeneration: disc herniation	
Lumbar degeneration: stenosis	
Acute spinal cord compression/contusion: degenerative	
Degenerative spine	
Spinal degeneration: other	
Spine Degenerative	
Thoracic degeneration: back pain	
Thoracic degeneration: disc herniation	
Thoracic degeneration: stenosis	
Back Pain	
Back pain	
Acute hydrocephalus: infection	Hydrocephalus
Acute hydrocephalus: intracranial haemorrhage	
Acute hydrocephalus: shunt in situ	
Acute hydrocephalus: tumour	
Chronic ventricular abnormality: no shunt	
Chronic ventricular abnormality: shunt in situ	
Hydrocephalus	
Hydrocephalus : other	
Shunt problem	
Shunt problem: other	

Specialist working diagnosis	Primary Classification
Intracranial infection	Infection or post-operative complication
Intracranial infection: other	
Intracranial infection: postoperative	
Ventricular/meningeal infection	
Acute hydrocephalus: infection	
Extra-axial infection	
Intracerebral infection	
Infections of the central nervous system	
Post-operative problem	
Post-operative problem : other	
Post-operative problem: other	
Post-operative problem: wound	
Post-operative problem: wound infection	
Post-operative wound problem	
Spinal Infection	
Spinal infection: epidural abscess only	
Spinal infection: intradural	
Spinal infection: other	
Spinal infection: paravertebral only	
Spinal infection: spondylodiscitis	
Spinal infection: postoperative	
Spinal fracture: C1	Spinal trauma
Spinal fracture: C2	
Spinal fracture	
Spinal fracture without trauma	
Spinal fracture: lumbar	
Spinal fracture: other	
Spinal fracture: sacrum	
Spinal fracture: thoracic	
Spinal fracture - traumatic	
Spinal Injury	
Spinal trauma: cauda equina injury	
Spinal trauma: cord injury	
Spinal trauma: disco-ligamentous injury only	
Stable spinal fractures: multiple levels	
Unstable spinal fractures: multiple levels	
Stable spinal fractures: multiple levels	
Spinal fracture: subaxial cervical (C3-C7)	
Spinal tumour: intradural	
Intracranial and intradural spinal tumours	
Spinal Tumour	
Spinal tumour: other	
Spinal tumour: primary extradural	
Metastatic Spinal Cord Compression	
spinal tumour including metastatic spinal cord compression	
Spinal tumour: metastases without cord/cauda equina compression	
Spinal tumour: metastatic spinal cord compression (MSCC)	
Spinal tumour: metastatic spinal cord compression (MSCC)	
Spinal tumour: metastases without cord/cauda equina compression	
Spinal tumour	
Intracerebral haemorrhage	Stroke
Ischaemic stroke	
Stroke	
Stroke / Vascular	
Spontaneous intracerebral haemorrhage or Ischaemic stroke	
Intraparenchymal or intraventircular Haemorrhage	Vascular
Intraventricular haemorrhage	
Intracerebral haemorrhage	
Neurovascular problem	
Neurovascular: other	
Spinal vascular problem	
Spontaneous subarachnoid haemorrhage	
Subarachnoid haemorrhage	
Spontaneous subarachnoid haemorrhage	
Not neurosurgical diagnosis	Not NS
Nothing abnormal detected	
Other	

Appendix 2. User feedback questionnaire with usability, acceptability and feasibility assessment.

Instrument	Stem	Item
Acceptability	Please rate the following statements according to the scale:<br>(1) Completely agree<br>(2) Somewhat disagree<br>(3) Neither agree nor disagree<br>(4) Somewhat agree<br>(5) Completely agree	The neurosurgical referral dashboard meets my approval
		The neurosurgical referral dashboard is appealing to me
		I like the neurosurgical dashboard
		I welcome the neurosurgical referral dashboard
Feasibility	Please rate the following statements according to the scale:<br>(1) Completely agree<br>(2) Somewhat disagree<br>(3) Neither agree nor disagree<br>(4) Somewhat agree<br>(5) Completely agree	The neurosurgical referral dashboard seems implementable
		Using the neurosurgical referral dashboard seems doable
		Using the neurosurgical referral dashboard seems possible
		The neurosurgical referral dashboard seems easy to use
Usability	Please rate the following statements according to the scale:<br>(1) Strongly disagree<br>(2) Somewhat disagree<br>(3) Neither agree nor disagree<br>(4) Somewhat agree<br>(5) Strongly agree	I think that I would like to use this dashboard frequently
		I found the dashboard unnecessarily complex
		I thought the dashboard was easy to use
		I think that I would need the support of a technical person to be able to use this dashboard
		I found the various functions in this dashboard were well integrated
		I thought there was too much inconsistency in this dashboard
		I would imagine that most people would learn to use this dashboard very quickly
		I found the dashboard very cumbersome to use
		I felt very confident using the dashboard
		I needed to learn a lot of things before I could get going with this dashboard
General	Which aspects or features of the dashboard did you find most useful?	
	Do you have any suggestions for improving the dashboard?	
	Which role would best describe you?<br>(1) Neurosurgical Registrar<br>(2) Neurosurgical Consultant<br>(3) Management and Administration	
